# Regulation of the NF-κB-Mediated Transcription of Inflammatory Genes

**DOI:** 10.3389/fimmu.2014.00071

**Published:** 2014-02-25

**Authors:** Dev Bhatt, Sankar Ghosh

**Affiliations:** ^1^Department of Microbiology and Immunology, College of Physicians and Surgeons, Columbia University, New York, NY, USA

**Keywords:** transcription, chromatin, gene expression, NF-kappaB, signaling, transcription factor

## Abstract

The NF-κB family of transcription factors plays a central role in the inducible expression of inflammatory genes during the immune response, and the proper regulation of these genes is a critical factor in the maintenance of immune homeostasis. The chromatin environment at stimulus-responsive NF-κB sites is a major determinant in transcription factor binding, and dynamic alteration of the chromatin state to facilitate transcription factor binding is a key regulatory mechanism. NF-κB is in turn able to influence the chromatin state through a variety of mechanisms, including the recruitment of chromatin modifying co-activator complexes such as p300, the competitive eviction of negative chromatin modifications, and the recruitment of components of the general transcriptional machinery. Frequently, the selective interaction with these co-activators is dependent on specific post-translational modification of NF-κB subunits. Finally, the mechanisms of inducible NF-κB activity in different immune cell types seem to be largely conserved. The diversity of cell-specific NF-κB-mediated transcriptional programs is established at the chromatin level during cell differentiation by lineage-defining transcription factors. These factors generate and maintain a cell-specific chromatin landscape that is accessible to NF-κB, thus restricting the inducible transcriptional response to a cell-appropriate output.

## Introduction

Upon pathogen detection, the innate immune system must be able to mount a robust and rapid response, but equally important is the need to rein in the cytotoxic effects of the inflammatory response. Therefore, modulation of pro-inflammatory gene expression is of fundamental importance in the maintenance of immune homeostasis. Pro-inflammatory genes are maintained in a silent, yet poised, state that can be rapidly induced in response to different stimuli, and this characteristic pattern is achieved through the action of two elements: the activation of inducible transcription factors and the modulation of the chromatin environment at gene regulatory elements.

Multiple signaling pathways are activated in response to immune and inflammatory stimuli, resulting in the activation of many different transcription factors. The transcription factors induced upon stimulation must interact with *cis*-regulatory elements of target genes to facilitate recruitment of the general transcriptional machinery. The chromatin state at these *cis*-elements plays a critical role in modulating the activity of transcription factors, mainly by functioning as a steric barrier to DNA binding and as a post-translational regulatory platform that influences the recruitment of transcriptional cofactors. This review will focus on the interplay of the archetypal inducible transcription factor NF-κB with the chromatin environment, and discuss how the chromatin presents a selective regulatory barrier to NF-κB activity and how NF-κB alters the chromatin environment to induce transcription of inflammatory genes.

The NF-κB family of transcription factors is conserved through metazoan organisms. These transcription factors are characterized by a unique DNA-binding motif known as the Rel homology domain (RHD). In mammals, there are five RHD containing proteins: p65 (RelA), c-Rel, RelB, p100/p50, and p105/p52. Each protein is capable of forming homodimers and heterodimers, with 15 dimer combinations possible (1). In unstimulated cells, NF-κB is sequestered in the cytoplasm by the inhibitor of NF-κB family (IκB) via their ankyrin repeat domains. Upon stimulation, the IκB kinase (IKK) complex is activated and phosphorylates serine residues on IκB molecules, targeting them for ubiquitination by the SCF E3 ligase and subsequent degradation by the proteasome. Degradation of IκB releases NF-κB, which translocates into the nucleus to initiate a transcriptional response (2).

### Basic transcriptional mechanisms

Transcription can fundamentally be understood as the interaction of *cis*- and *trans*-acting factors within the nucleus that orchestrate the expression of a particular gene. The *cis*-acting elements are defined as non-coding DNA elements located on the same chromosome as the protein-coding locus. Two critical *cis*-elements are the promoter and the enhancer. Trans-acting factors include sequence-specific transcription factors (such as NF-κB and IRF), chromatin modifying complexes (such as histone acetyltransferases and chromatin remodeling complexes), the mediator complex, and finally general transcriptional factors (GTFs), including RNA polymerase II (3–5).

Transcription initiates from the *cis*-regulatory element known as the promoter, which can be divided into two portions, the core promoter and the proximal promoter. The core promoter contains regulatory elements that bind Pol II and the general transcriptional machinery, extending from −35 to +35 relative to the transcriptional start site. The distal promoter extends ~300 bp upstream from the core promoter and serves as the binding site for sequence-specific transcription factors. It is thought that proximal promoter binding transcription factors coordinate with distal enhancer-binding transcription factors to recruit the mediator complex and the general transcriptional machinery.

A number of conserved elements comprise the core promoter. This includes, but is not limited to, the BRE (TFIIB recognition element), TATA box, initiator, and downstream promoter element (DPE). However, not all of these elements are absolutely required for a promoter to be functional. In addition to these discrete elements, vertebrate promoters are also distinguished by their CpG dinucleotide content, with high CpG content promoters commonly classified as CpG-island promoters (CGI) (6–8). However, all these regulatory elements function within the context of chromatin.

Eukaryotic DNA is highly condensed, a necessary strategy for the compaction of large genomes into a relatively small nuclear volume. This is achieved by the wrapping of DNA around histone proteins. The resultant DNA:histone complex is referred to as the nucleosome, which is the core repeating unit of the chromatin. The degree of compaction of chromatin fibers plays an important role in accessibility of DNA-binding proteins for their cognate binding sites. Furthermore, elongating polymerases encounter nucleosomal barriers during their progression through the locus. Thus, chromatin presents additional layers of complexity to the mechanism of transcription (9).

There are four major histone proteins, H2A, H2b, H3, and H4. Two copies of each protein form the histone octamer, and 147 bp of DNA wraps around the octamer to form the nucleosome. The interaction of DNA and the octamer is highly stable. High-resolution crystal structures have shown that there are over 100 points of contact between the octamer and DNA, and that the DNA is stabilized by arginine residues of the octamer directly contacting the minor groove of DNA (10).

### Histones and chromatin modification domains at regulatory elements

The tails of histone proteins are subject to a broad range of post-translational modifications that influence all aspects of chromatin biology. Histone modification is thought to have two major purposes: (1) the alteration of net charge of the tail, which has an effect on tail-DNA interactions and inter-histone interactions; (2) the generation of recognition sites for activating or repressive factors (9).

Lysine acetylation and methylation have been the most well-studied histone modifications that regulate transcription. Addition of acetyl residues is accomplished by histone acetyltransferase complexes (HATs), and removal of these marks is catalyzed by histone deacetylases (HDACs). HATs, such as the p300/CBP complex, are generally non-specific and are able to target multiple lysine residues on various histone proteins. Lysine acetylation is largely associated with active transcription. Acetylation of H4K16 has been shown to have a significant impact on the compaction of nucleosomal arrays *in vitro*, and is a general mark of euchromatin *in vivo*. The specialized domain that recognizes acetylated lysines is known as the bromodomain. Proteins that have bromodomains include HATs and chromatin remodeling complexes such as RSC and SWI/SNF (11, 12).

Lysine methylation can act as an activating or repressive mark, depending on the specific residue modified. For example, trimethylation of H3K4 is strongly associated with active transcription, and is found at active promoters, while methylation of H3K9 and H3K27 is associated with transcriptional repression. In contrast to acetyltransferases, methyltransferase have very restricted substrate specificities, in keeping with the more specialized and context-dependent roles that methylated lysines have. Furthermore, methylated lysine residues have relatively restricted distributions across gene loci; for example, H3K4me3 is enriched at promoters of active genes, while H3K4me1 has been more recently recognized as an enhancer-specific mark, and finally H3K36me3 and H3K79me are found within gene bodies (3, 11).

### Setting the stage: Developmental establishment of an NF-κB-responsive gene program

NF-κB dimers are broadly distributed, especially in cells of the immune system, and are activated in response to a variety of cell-specific receptor stimuli (e.g., TCR in T-cells, pattern recognition receptors in myeloid cells). In response to these stimuli however, NF-κB is able to induce the expression of a diverse range of genes tailored to a specific cellular response. Such cell-type specific response is established during development and is broadly speaking, the result of lineage-defining transcription factors binding to enhancer elements at an early developmental stage. In macrophages for example, the major lineage-determining transcription factors PU.1 and C/EBP and AP-1 families bind cognate enhancer elements and establish an active chromatin state. This renders the enhancer and promoter elements accessible to additional factors, resulting in a cell-specific array of binding sites in genes that have been epigenetically primed for binding to activated NF-κB (13, 14). The establishment of an inducible epigenetic landscape is not the only mechanism by which NF-κB binding is modulated. Many loci require the synergistic activity of multiple signal-dependent transcription factors, as well as further modulation of the chromatin structure at enhancers and promoter elements.

### Regulation before the signal: Chromatin as a determinant for NF-κB binding activity

The concerted activity of lineage-defining transcription factors and inducible transcription factors are required for the proper regulation of inflammatory genes expression. Along those lines, the synergistic activity of multiple inducible transcription factors is frequently necessary to overcome the inhibitory chromatin state at inflammatory genes. A classic example of transcription factor synergism is the induction of the human interferon-β (*IFNB*) gene in response to viral infection. Stimulus-dependent expression of this gene requires the cooperative binding of three transcription factors: NF-κB, IRF3/IRF7, and ATF-2/c-JUN. NF-κB initially binds to the conserved PRDII element in the promoter. This in turn facilitates the recruitment of IRF and ATF-2/c-Jun. Once properly assembled at the promoter, these transcription factors serve as a platform for the sequential recruitment of the PCAF chromatin modifying complex, the p300/CBP acetyltransferase, and subsequently the SWI/SNF chromatin remodeling complexes. SWI/SNF remodels the downstream nucleosome that encompasses the TATA box, thus allowing TBP binding and subsequent pre-initiation complex assembly (15–17). The regulation of the IFNβ enhanceosome is an important illustration of transcriptional regulation through the combinatorial control of multiple transcriptional factors, and how only the concerted effort of these factors can resolve the presence of a chromatin barrier.

Studies on the regulation of expression of the cytokine gene *Il12b* have shown that a non-permissive chromatin configuration functions as regulatory barrier that must be inducibly resolved for proper gene expression. Early studies on *Il12b* expression focused on identifying critical transcription factor binding sites by systematic mutation of promoter–reporter constructs. These studies showed that NF-κB, specifically the c-Rel subunit, in conjunction with C/EBP, AP-1, and NFAT transcription factors, are required for *Il12b* activation (18–21). Nucleosomal mapping of endogenous *Il12b* promoter revealed that the critical transcription factor binding sites were occupied by a positioned nucleosome extending from −30 to −175 upstream of the transcriptional initiation site, and that this nucleosome was selectively remodeled upon LPS stimulation. Furthermore, this promoter remodeling event was dependent on *de novo* protein synthesis, but was independent of c-Rel binding, as evidenced by promoter remodeling occurring in c-Rel deficient cells that lacked the ability to express *Il12b* (22–24). Thus, in contrast to the enhanceosome, which required NF-κB in a synergistic complex to recruit chromatin remodeling complexes, *Il12b* operates under a slightly different mode of regulation in which remodeling is required prior to DNA binding. Moreover, since NF-κB genes are induced with variable kinetics, there would appear to be multiple chromatin-based regulatory strategies governing the binding of NF-κB.

To this end, Saccani and colleagues made the important observation that although NF-κB RelA subunits entered the nucleus rapidly upon stimulation, they only bound to a subset of genes initially (25). Other genes were bound at later time points, suggesting that their binding sites were inaccessible, consistent with the observations at the *Il12b* promoter. Inspection of the chromatin modifications at early versus late bound genes showed a consistent pattern: early recruiting genes had higher levels of histone acetylation while late bound genes had low basal acetylation levels that increased upon stimulation (25). From these data, a model emerged in which NF-κB binding was inhibited at certain promoters during the early phase of stimulation by inaccessible chromatin structure, and NF-κB could only bind these late gene promoters after critical chromatin remodeling events had occurred.

Implicit in this model is the idea that NF-κB itself lacks the ability to bind a chromatinized template and requires the binding of additional factors capable of recruiting chromatin remodeling complexes. This hypothesis is supported by structural studies of NF-κB, which showed its precise binding to a naked DNA template (26–28). It has been reported that NF-κB p50-homodimers can indeed bind nucleosomal κB sites *in vitro*, albeit at a reduced efficiency than the naked template (29, 30). Furthermore, the positioning of the binding site within the nucleosome strongly influenced the binding affinity of p50, with binding sites near the edge of the nucleosome being highly favored. The *in vitro* nucleosome binding was also at least partially dependent on remodeling complex activity or partial disassembly of the histone octamer (31). It remains to be seen whether this nucleosome binding activity is specific to p50-homodimers and its specific cognate site, or whether it is a common feature shared by a broad variety of NF-κB dimers and binding sites. Interestingly, p50 dimer complexes have been observed in the nucleus of unstimulated cells, which are displaced by activating dimers (RelA or c-Rel species) in stimulated cells presumably after chromatin remodeling has occurred (32, 33). Given that p50 lacks a trans-activation domain, and has been shown to associate with deacetylase complexes, the latent p50 binding to a more compact chromatin template may be part of a regulatory strategy keeping genes silent under resting conditions by maintaining a repressive chromatin environment (32).

Although it is clear that differential chromatin states influence the NF-κB response, the mechanisms that contribute to inducible chromatin remodeling remained unclear. To test whether specific chromatin remodeling complexes were required for expression of NF-κB dependent genes, Ramirez-Carrozzi and colleagues targeted the SWI/SNF chromatin remodeling complex by retroviral knockdown of its ATPase subunits, Brg and Brm (34). This protein complex was a likely candidate for chromatin remodeling as it had been shown to be strongly associated with gene activation in various contexts. In the targeted cells, the chromatin remodeling of *Il12b* was strongly inhibited, concurrent with a loss in expression of *Il12b*. The knockdown, however, had no effect on the inducible expression of another gene, the rapidly expressed chemokine, *Cxcl2*. Further comparison of the expression patterns of these two genes revealed that (1) *Cxcl2* was strongly induced upon 30 min of LPS treatment while *Il12b* was most strongly induced only after 2 h of treatment and (2) expression of *Cxcl2* was not inhibited by protein synthesis inhibitors, indicating that it is a primary response gene. Based on such criteria including induction kinetics, protein synthesis requirement, and SWI/SNF dependence, inducible genes could be partitioned into three classes: early primary, late primary, and secondary. The early primary response class was induced rapidly and did not require either Brg1/Brm or new protein synthesis and included genes such as *Cxcl2, Tnf*, and *Ptgs2*. However, a subset of primary response genes (which included *Ccl5, Saa3*, and *Ifnb1*) did require Brg1/Brm for activation and were induced with delayed kinetics relative to remodeling-independent primary response genes. Finally, secondary response class (which included *Il12b, Nos2*, and *Il6*) required both Brg1/Brm and new protein synthesis. ChIP analysis of Brg showed that the remodeling complex was associated in an inducible fashion at remodeling-dependent genes. Furthermore, nuclease sensitivity analysis at representative genes from each class showed a pattern consistent with their dependence on Brg1/Brm. Namely, inducible promoter accessibility was seen at late primary and secondary genes, and this sensitivity was lost during knockdown of Brg1/Brm. In contrast, early primary genes had much more accessible promoters and this accessibility was unchanged by Brg1/Brm knockdown (34, 35).

This classification of inflammatory genes was further expanded by the discovery that many remodeling-independent genes were distinguished by the presence of a CpG island (CGIs) (35, 36). CGIs are prevalent in the promoters of ~70% of protein-coding gene promoters, including the promoters of ubiquitously expressed housekeeping genes (37). The presence of CpG-island promoters is highly correlated with an accessible chromatin configuration, namely high levels of H3K4me3 marks, as well as pre-association of Pol II in the basal state. The contribution CGIs to this favorable chromatin state is thought to emerge from multiple possible mechanisms. First, the recruitment of chromatin modifying complexes that deposit H3K4m3 marks and display specificity for CGIs via CXXC domain containing proteins (38, 39). Secondly, CGI sequences may be inherently unfavorable to the formation of stable, repressive nucleosome formation at these promoters (35, 40). Finally, many transcription factors, such as Sp1, have binding affinity for GC-rich sequences, and may contribute to the maintenance of an open chromatin configuration of CGI promoters. In contrast, non-CpG-island genes were largely remodeling dependent, and many such genes have been shown to be dependent on the IRF transcription factors, which are competent for recruiting SWI/SNF complexes.

**Figure 1:**
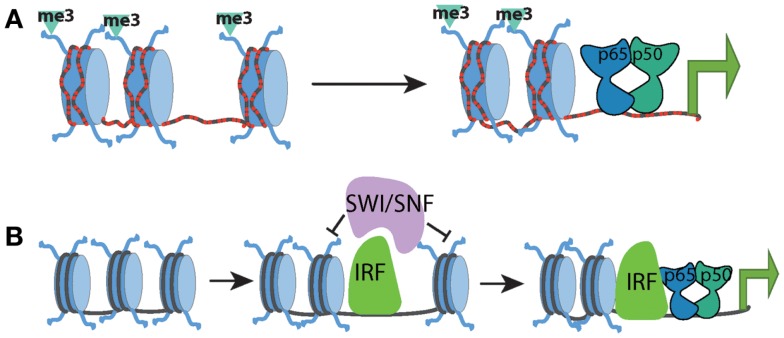
**Chromatin state at different promoter classes dictates the kinetics of the NF-kB response**. **(A)** CpG-island promoters (red dashed lines) are characterized by high levels of histone H3K4me3 and more highly accessible chromatin. This allows for rapid transcriptional induction by NF-kB, independent of the need for any SWI/SNF-dependent nucleosome remodeling. **(B)** Low-CpG promoters contain inactive and inaccessible chromatin signatures in the basal state, which forms a barrier to NF-kB binding and transcriptional activation. SWI/SNF complexes must be recruited to these genes by additional factors such as IRF proteins in order to facilitate chromatin remodeling. Upon chromatin remodeling, NF-kB can bind and induce transcription.

These studies have generated a regulatory framework that integrates the basal chromatin state with the kinetics of transcriptional induction and selective requirement for chromatin remodeling. Within this model, the kinetics of a particular gene’s expression correlated with the basal chromatin state and the synthesis and induction of transcription factors (such as IRF) capable of promoting the remodeling of nucleosomal barriers to NF-κB dimer recruitment and transcriptional activation. NF-κB dimers may still be involved in recruitment of remodeling complexes but are most likely to do so in cooperation with other transcription factors (Figure 1).

Subsequent to binding, NF-κB plays a major, yet heterogeneous role, at the chromatin level. The many different interactions between NF-κB and its various transcriptional cofactors have not been extensively defined, and the molecular order of many of these various interactions remain unclear. Nevertheless, it is accepted that NF-κB regulates the expression of its target genes through a broad range of chromatin-mediated mechanisms. These roles can be generalized into two broadly defined activities: recruitment of positive cofactors/marks and the removal of negative regulators/marks, as discussed below.

## NF-κB in the Nucleus: A Variety of Roles and a Variety of Partners

### Recruitment of co-activators

NF-κB is capable of interacting with many different transcriptional cofactors via its trans-activation domain, including chromatin modifying complexes and general transcription factors (41). The purpose of these chromatin modifications is to serve as a recruitment platform for additional activators, such as remodeling complexes, enabling recruitment of the transcriptional machinery to the promoter, followed by initiation of transcription. Although many of these cofactors are essential for the expression of NF-κB-induced genes, the mechanisms of how NF-κB can selectively regulate or recruit them remains unclear.

One of the most well-characterized cofactors of NF-κB, specifically the RelA subunit, is the p300/CBP histone acetyltransferase complex. The interaction between these factors is interesting in that it requires additional modification of RelA in order to occur. Post-translational modifications of RelA have been shown to play an important role in modulating the activity of NF-κB. Although the mechanistic importance of many of these modifications remains unclear, studies of specific residues have shown that they may regulate the recruitment of transcriptional cofactors such as histone acetyltransferases (HATs) or histone deacetylases (HDACs). The p300/CBP complex is an important example of a chromatin modifying complex that directly interacts with NF-κB. Strikingly this interaction is regulated at the post-translational level, being dependent on the specific phosphorylation of the RelA protein.

**Figure 2:**
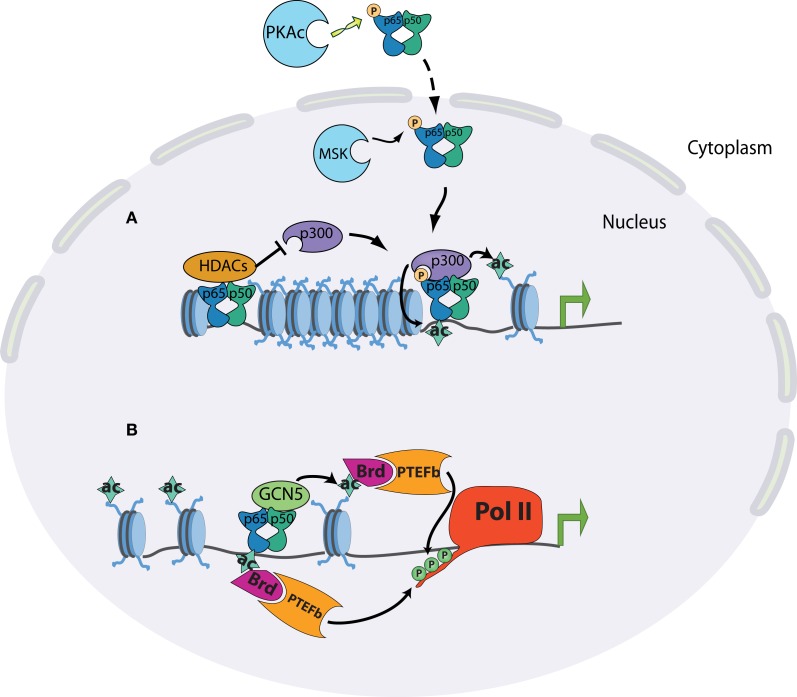
**Chromatin state at different promoter classes dictates the kinetics of the NF-kB response**. **(A)** Release of NF-kB dimers through appropriate stimulation is linked to phosphorylation of p65 at serine 276 in the cytoplasm by PKAc or in the nucleus by MSK1/2. Phosphorylated p65 preferentially interacts with CBP/p300 co-activator complexes, displacing its latent binding to HDAC complexes. The specific interaction of p65 and p300 results in the acetylation of p65 itself and of surrounding histones, and subsequent transcriptional induction. **(B)** The acetylation of p65 on K310 promotes the recruitment of the positive elongation factors Brd4 and P-TEFb, which can phosphorylate the polymerase C-terminus. p65 can also recruit GCN5 acetyltransferase complexes, leading to promoter acetylation, Brd4-P-TEFb recruitment, and polymerase elongation.

The Ser276 residue of RelA can be phosphorylated, has been shown to be targeted by the catalytic subunit of protein kinase A (PKA), mitogen- and stress-activated protein kinase-1 (MSK1), and Pim1 kinase (42–45). The importance of this phosphorylated serine was shown to be threefold: it moderately enhanced DNA-binding activity of RelA; it caused a conformational shift that allowed CBP/p300 binding in place of the latent interaction of RelA with HDAC complexes; RelA deficient cells reconstituted with S276A mutants were severely impaired in NF-κB dependent gene expression (32, 45–48) (Figure 2). Most convincingly, the absolute necessity of this phosphorylation event was demonstrated by the generation of a knock-in mouse containing an alanine substitution at RelA S276 residue. Mice homozygous for S276A point mutants were embryonic lethal between E11 and E16 and exhibited severe developmental defects, most notably in eye development, and embryonic fibroblasts showed a defect in the activation of selective subsets of pro-inflammatory genes (47).

The interplay between p300/CBP and RelA illustrates critical aspects of NF-κB biology. In order for NF-κB to fully function, it must be activated by canonical NF-κB signaling, and then modified by a distinct signaling pathway. p300/CBP has also been shown to acetylate RelA itself, notably on lysine 310. As with phosphorylation of Ser276, differential modification of Lys310 plays a major role in dictating RelA cofactor specificity. Acetylation of the residue promotes an interaction with the histone acetyltransferase Tip60 and the transcriptional elongation factors PTEF-B (49–51). Conversely, methylation of Lys310 by the SET6 proteins facilitates an interaction with the histone methyltransferase GLP/G9a, leading to downregulation of transcription. The selective modulation of these two residues highlights the functional diversity of NF-κB in the control of the inflammatory response. Many other post-translational modifications have been shown to occur on RelA, with a corresponding enhancement or attenuation of transcription of selective inflammatory genes (52, 53).

### De-repression: Removal of repressive marks and complexes

In addition to the inducible recruitment of activating marks, another regulatory mechanism is to maintain a basal repressive state at gene promoters. These repressive marks are subsequently removed upon stimulation. This phenomenon can occur at the level of basal chromatin marks, or the binding of repressive complexes. For example, some inducible gene promoters have high levels of the H3K9 dimethyl modification, a repressive mark associated with transcriptional silencing. Upon stimulation, this mark is removed by the Aof1 histone demethylase, which is recruited by initially bound c-Rel dimers (54, 55). Similarly, subsets of inflammatory gene promoters are marked by the repressive trimethyl H3K27 deposited by the Ezh2 methyltransferase, and the inducible removal of this mark by the Jmjd3 demethylase is required for their expression (56). The dynamic regulation of this mark has proved to be of interest, due to the development of a Jmjd3-specific pharmacological inhibitor that can attenuate inducible pro-inflammatory gene expression (57).

The stimulus-dependent eviction of basally bound co-repressor complexes at promoters is another chromatin-based strategy for maintaining tight regulation on the expression of NF-κB dependent genes. Nuclear receptor corepressor (NCoR) and the closely related protein silencing mediator of retinoid and thyroid receptors (SMRT) are multiprotein co-repressor complexes containing histone deacetylases (HDACs), specifically HDAC3. These HDACs function to keep H3K9/14 acetylation levels low in the resting cell. Upon stimulation, this repressive state is relieved, allowing for the accumulation of activating marks and recruitment of the transcriptional machinery. Similarly, NCoR/SMRT complexes are also displaced, promoting NF-κB binding and recruitment of co-activator complexes. It should be noted that both NCoR and SMRT complexes function in non-redundant fashion, and are recruited to regulatory elements by different sequence-specific transcription factors: JUN homodimers and p50 dimers in the case of NCoR, and the ETS-related protein, translocation–ETS–leukemia (TEL) in the case of SMRT (36, 58, 59). In addition to HDAC-dependent removal of activating marks, NCoR complexes can maintain a silent state via the deposition of the repressive H3K20me3 mark. Subsequently upon stimulation, these marks are removed by the NF-κB dependent recruitment of histone demethylase (60).

Because these repressive complexes restrain the expression of inflammatory gene program in the basal state, one would predict that their targeted disruption would lead to hyperacetylation and therefore enhanced expression of immune genes. Surprisingly however this is not the case. Knockout of NCoR in macrophages led to an anti-inflammatory phenotype, contrary to the expectation that removal of a basal repressor would lead to the hyper-responsiveness of pro-inflammatory genes. Although NF-κB binding activity appeared to be unaffected in these cells, the lack of the basal repressive complex compromised the stimulus-dependent deposition of H3K4me2, a mark associated with productive transcription. The mechanism contributing to this phenotype was attributed to the de-repression of metabolic pathways that subsequently inhibit chromatin modifying complexes required for induction of pro-inflammatory genes (61). Along similar lines, HDAC3-deficient macrophages were defective in their ability to activate a broad range of pro-inflammatory genes. Although the knockout did result in hyperacetylation across immune genes, there was a specific defect in the expression of IFNβ, which compromised overall STAT protein signaling and the secondary gene response to TLR4 stimulation (62).

### NF-κB-mediated regulation of Pol II elongation

After polymerase recruitment to the promoter, sequential modifications of Pol II are required for productive transcription to occur. The initial stage of transcriptional elongation requires local unwinding of the promoter DNA. Characteristic of this stage is the phosphorylation of serine 5 of the C-terminal heptad repeat by the Cdk7 subunit of TFIIH, or the CDK8 subunit of the mediator complex. Ser5 phosphorylation serves as a platform to recruit RNA capping enzymes to the nascent transcript (63, 64). A second pause occurs ~40–50 bp downstream of the transcription initiation site, and de-repression of this block requires the kinase activity of P-TEFb. This final de-repression commits the polymerase to the elongation phase of transcription (65, 66). During an inflammatory response, there is evidence for stimulus-dependent unpausing being the major rate-limiting step in the expression of rapidly induced genes (36, 67, 68). Along with the p300/CBP complex, NF-κB has been shown to recruit GCN5 acetyltransferase complexes, which primarily modify H4K5/K8/K12 lysines. These residues have been shown to be deposited in response to NF-κB binding. The accumulation of acetylated H4 in stimulated cells allows binding of the bromodomain-containing protein BRD4, which then plays a positive role in transcription by recruiting the elongation factor P-TEFb (Figure 2) (36).

The signal-dependent elongation by Pol II is most prevalent at rapidly induced CGI containing promoters, where the permissive chromatin structure allows for a pre-assembled polymerase complex to exist in the basal state. At these genes, the major regulatory checkpoint is therefore the licensing of the polymerase to enter a productive elongation phase, as opposed to the recruitment of polymerase itself. Signal-dependent elongation is a well-conserved regulatory mechanism, having been well-documented at the heat-shock genes, and more recently at rapidly induced immune genes in *Drosophila* (69, 70). The inhibition of BRD4 by highly selective chemical compounds, however, revealed that such an inhibition can selectively inhibit low-CpG containing genes (71). Although BRD4 activity is essential for a broad class of NF-κB dependent genes, the selective effect of the I-BET chemical antagonists implies multiple mechanisms of BRD4 recruitment. Indeed, there is evidence that acetylated NF-κB can directly recruit BRD4 (72).

## Chromatin Dynamics at Enhancer Regions: A Mechanism for Immunological Memory and Variability?

Chromatin dynamics at promoters play a central role in how inducible genes are regulated, but advances in genome-wide studies have shed new light on how enhancer regions can also play a dynamic regulatory role. Because enhancer elements can be located great distances from the transcriptional start sites (ranging from a few kilobases to over a megabase), identification of bona fide enhancers has been a significant challenge, and the mechanisms of enhancer-mediated regulation of transcription remain somewhat unclear. As discussed above, enhancers are important in establishing the transcriptional competence of a locus following the binding of lineage-specifying factors. A highly multiplexed ChIP-seq study by Garber et al. in dendritic cells further developed this model, differentiating enhancer-binding transcription factors into three major regulatory classes: stably bound pioneer factors C/EBP and PU.1, which presumably bind to inaccessible chromatin early in lineage commitment; “primer” factors, such as Jun and AP-1, which are stably bound subsequent to pioneer factor binding and presumably contribute to local chromatin modification and remodeling; and finally the inducible factors, chiefly NF-κB and STAT proteins (73). Concurrent with inducible transcription factor binding, enhancer chromatin modifications are also dynamic. Active enhancer regions in a broad variety of cell types are distinguished by high levels of H3K4 monomethylation, and a number of ChIP-seq studies have utilized this specific mark to discover novel enhancers at a genome-wide level (74, 75).

The H3K27 acetyl mark has recently been demonstrated as another important enhancer-associated modification (76). Genome-wide analyses of this mark in macrophages has enabled functional categorization of enhancer elements into constitutive, poised, latent, and repressed classes based on the dynamics of the two histone modifications and PU.1 binding. Constitutively active enhancers are marked by PU.1 binding and high levels of both H3K4m1 and H3K27ac that remain stable upon stimulation. Poised enhancers only contain PU.1 and H3Km1, and H3K27 is dynamically acetylated upon stimulation. Latent enhancers are devoid of these marks, which are deposited upon stimulation. These enhancers govern the expression of late-phase genes, and are established in a stimulus-specific manner. Specifically, activation via TNF and IL1β, potent activators of the NF-κB signaling pathway, results in latent enhancers that are bound by the lineage definer PU.1 and NF-κB. By contrast, interferon stimulation results in the appearance of a latent enhancer repertoire enriched for PU.1 and STAT/IRF binding sites. In this manner, cells that have been exposed to a specific stimulus retain a short-term epigenetic memory of that stimulus, facilitating a more rapid and efficient transcriptional response upon subsequent stimulation (77). On the other hand, repressive chromatin modifications can be deposited at regulatory elements upon an initial stimulus, thereby attenuating responses to a secondary stimulus, and causing the cell to become tolerized against the stimulus. At the level of chromatin, it has been shown that promoters of tolerizable genes become hypoacetylated and adopt an inaccessible chromatin structure and are subsequently hyporesponsive (78).

Given that enhancers play such an important modulatory role in the inducible gene program, there has been considerable interest in how genetic variation of regulatory elements can influence gene expression. Heinz and colleagues recently showed that strain-specific genetic variations at enhancers affected PU.1 and CEB/P binding in macrophages. Loss or gain of these lineage-determining factors was concomitant with alterations of H3Km1 and H3K27ac levels, as well as the expression levels of nearby genes. Importantly, the variability in lineage-factor binding sites resulted in a loss of NF-κB binding at enhancers, much more so than variation of κB sites themselves (79). The strong correlation between loss of lineage-factor binding, loss of chromatin modification, and loss of NF-κB, support the role of NF-κB as a multifunctional switch that can only bind to sites marked and poised by lineage-defining factors.

## Future Directions and Advances

In the 25 years since its discovery, NF-κB has become one of the most heavily studied transcription factors. This is of course not without cause, as it is well-appreciated that a number of disease states, particularly inflammatory disease states, are at some level due to dysregulated NF-κB signaling. This review has attempted to summarize the broad range of chromatin-regulated mechanisms that govern the specificity of NF-κB-mediated transcription, the variety of ways NF-κB itself can influence chromatin structure, and a survey of how epigenetic programs initiated in response to NF-κB are established and propagated. A recurring theme in the study of NF-κB is that this transcription factor rarely acts alone. Lineage-determining transcription factors set the stage at the chromatin level, thereby dictating the response NF-κB will induce. Furthermore, a number of chromatin changes must take place at NF-κB dependent genes in order for proper gene induction to occur. In this sense, NF-κB can be thought of as the final regulatory switch. At rapidly induced genes, the switch controls late events such as the licensing of a pre-assembled polymerase into a productive elongation phase. In late-expressed genes, chromatin remodeling must occur, polymerase must be recruited and transition into an elongation phase takes place. This regulatory scheme largely focuses on events at the promoter, and indeed only fairly recently have the mechanisms of enhancer regulation been appreciated. However, a number of outstanding questions remain to be answered. Post-translational modification of NF-κB has long been appreciated as an important regulatory mechanism, dictating cofactor specificity for active dimers, but the physiological necessity of many of these modifications remains unclear outside of *in vitro* settings. In many of these cases, the identity of modification-dependent interaction partners is unknown, and the spatiotemporal regulation of the majority of modifications remains unclear. Furthermore, the relative contributions of the multiple regulatory mechanisms remain unclear. For example, the p300/CBP bound by NF-κB plays a role in enhancing transcription of inducible genes (as in the case of the IFNβ enhanceosome), but its direct acetylation of NF-κB also plays a critical and perhaps more diverse regulatory role. It is possible that NF-κB cofactor requirements are dictated by cooperative transcription factor binding at specific regulatory elements. As with the case of the RelA Ser276 residue, generation of genetic models targeted at specific residues would be of tremendous value.

Genome-wide studies of the chromatin state during a pathogenic response have strengthened the understanding of the various regulatory mechanisms involved in establishing a competent transcriptional response. Although our understanding of the dynamics of NF-κB and its relationship with lineage-defining factors has deepened, it should be noted that there are numerous transcription factors and chromatin modifying complexes whose roles and relationship with NF-κB remain to be further characterized. Integrative and multiplexed studies have given us a snapshot of the different hierarchies of transcription factor binding (73), and similar studies examining the panoply of chromatin modifications will likely prove fruitful as well. By systematically targeting different chromatin modifiers, either chemically or genetically, a deeper understanding of the regulatory logic governing the NF-κB transcriptional response can be developed.

## Conflict of Interest Statement

The authors declare that the research was conducted in the absence of any commercial or financial relationships that could be construed as a potential conflict of interest.
